# Potential Use of Triethylenediamine (TETA)-Cured Epoxy Resin in Cemented Soil for Slope Protection and Restoration

**DOI:** 10.3390/ma19091735

**Published:** 2026-04-24

**Authors:** Yifan Xue, Ping Lyu, Wei Wu, Hui Zeng, Fengwei Xing, Xiaoteng Li, Hongqiang Chu, Fengchen Zhang

**Affiliations:** 1 College of Civil and Transport Engineering, Hohai University, Nanjing 210098, China; 2 College of Materials Science and Engineering, Hohai University, Changzhou 213000, China; 3 Department of Civil, Construction and Environmental Engineering, Iowa State University, Ames, IA 50011, USA; 4 China Yangtze Power Co., Ltd., Yichang 443002, China; 5 Department of Civil Engineering, Neapolis University Pafos, Pafos 8042, Cyprus

**Keywords:** cementitious binders, soil stabilization, triethylenediamine-cured epoxy resin, pore structure characteristics, slope restoration

## Abstract

With the requirement for reducing carbon footprint in engineering construction, porous vegetation concrete is increasingly receiving attention for use in completed slope restoration. Cemented soil is introduced after the completion of porous vegetation concrete stabilization and functions mainly as a revegetation substrate. An important consideration for cemented soil in this application is its ability to maintain strength and water stability and possess moisture retention capacity, without causing much increase in alkali release or diffusion. This present study investigated a newly developed twofold stabilization system involving both cement binders and organic waterborne epoxy resin to meet the requirements of synthetically enhancing slope stabilization and restoration. Changes in the unconfined compressive strength and water stability were analyzed, whilst mineralogical composition and microstructure characteristics were investigated. The results indicated that moderate incorporation of triethylenediamine (TETA)-cured epoxy resin (1–2% by soil mass) moderately reduced strength and increased water stability with controlled alkali release in cemented soil. Mineralogical and microstructural analysis revealed that TETA-cured epoxy resin retarded cement hydration and refined particle bonding, exhibiting less consolidated pore structure characteristics. The twofold stabilization was exceptional in enhancing structural stability exposed to repeated humidity variation, albeit it yielded increased strength reduction rate from <7% to 9–16% under UV irradiation. Potentials of calcium sulfoaluminate cement and Portland slag cement were also investigated. A pilot-scale vegetation trial with representative plant species gave general agreement with effects observed in the laboratory in alkali reduction and moisture retention. The results provided an ecological approach for better restoring completed slopes that were stabilized using porous vegetation concrete.

## 1. Introduction

Concrete stabilization has been widely conducted in engineering constructions such as riparian embankment protection, rock-cut slope coverage, and abandoned mines restoration [1]. However, the use of traditional concretes leaves these completed slopes inappropriate for rehabilitation and disturbs ecological biodiversity [2,3,4]. Nowadays, porous vegetation concrete is developed as a more sustainable construction material, especially for slope stabilization and restoration [5]. Due to its designated high porosity, porous vegetation concrete has significant advantages in carbon footprint reduction and environmental protection. Typically, the cement consumption for porous vegetation concrete is only 50–70% of that of conventional ordinary concrete, and this contributes to a CO_2_ emission decrease by 0.5–0.7 t/m^3^ [3,6]. Additionally, due to the biochemical respiration of plants that can be successfully revegetated, approximately 4–20 kg/m^2^ of additional CO_2_ can be sequestrated annually [7]. However, challenges remain regarding porous vegetation concrete, providing instant protection and facilitating biocompatibility.

Typically, porous vegetation concrete comprises cement and specially graded aggregates that generally involve no fine aggregate of sand [8]. This is intended for yielding a concrete structure that consists of connected internal pores and void channels. These pores and voids are expected to provide rational spaces for the establishment of vegetation roots and subsequent propagation with appropriate anchoring [9,10,11]. In order to achieve exceptional biocompatibility, these voids in porous vegetation concrete generally require replenishment with planting substrates of, for instance, humus soil [5]. There are typically two types of planting structures for porous vegetation concrete, clarified according to their sowing position, namely, the exterior layer-covering type and interior grouting type. For exterior layer-covering, the completed porous vegetation concrete for slope stabilization is overlaid with planting substrate as a vegetation layer. Alternatively, the substrate, premixed with plant seeds and likely artificial nutrients, can be injected into the concrete voids. However, both types require the planting substrate to possess sufficient strength and resistance to water erosion in combination with adequate moisture retention. Moreover, the introduced planting substrate can be susceptible to the high alkalinity of concrete induced by the release of alkalis from Ca(OH)_2_ [12,13], which can severely damage seedlings’ survival. Therefore, it is necessary to introduce appropriate stabilization methods to the soil for application scenarios of porous vegetation concrete in achieving green-growing.

Generally, soil stabilization has been widely utilized in various engineering applications for its superior solidification capacity and rapid strength development [14,15,16]. Chemical stabilization of soil can be achieved by incorporating additives such as cementitious binders, organic polymers, as well as bituminous materials. However, when it comes to ecologically restoring concrete slopes, cemented soil confronts the disadvantages of physical degradation and distortion of the soil, reduced moisture retention, and increased alkali diffusion [17,18,19]. To address these limitations, research efforts are devoted to proposing improved modifications. Strategies predominantly rely on incorporating supplementary cementitious materials such as fly ash, granulated blast furnace slag, silica fume, among others, to mitigate the alkali concentration impact induced from Portland cement hydration on disturbing biological activity [20,21,22,23]. Alternatively, novel materials of nano-MgO, graphene oxide, polypropylene fibers, etc., are also investigated to mainly refine the structural characteristics [17,24,25,26,27]. However, these mineral or fiber additives contribute to nearly no changes in the moisture retention potentials for stabilized soil, and consequently, they are not ideal for the modification of cemented soil that is used as a backfilled planting substrate in porous vegetation concrete.

Alternatively, organic polymer modification exhibits promising adaptability in cemented soil for providing controlled alkali release and improved moisture retention [28,29,30,31]. Recent studies manifested that waterborne epoxy resin can refine microstructures of composites and enhance macroscopic performance through mechanisms of electrostatic attraction and cross-linking between particles [32,33,34]. Reported studies on waterborne epoxy resin modification, to the best of the authors’ knowledge, have primarily focused on asphalt mixtures, road repair materials, and waterproofing, leakage-prevention applications. For instance, Han et al. investigated mixing waterborne epoxy resin with superfine Portland-sulphoaluminate cement and found that the average water penetration height of the waterborne epoxy resin-modified material was only 52.2% that of the cementitious materials, indicating better impermeability performance whilst when 3% waterborne epoxy resin was mixed, the bending strength of the composite was 10.6% higher [35]. However, applications of waterborne epoxy resin in cemented soil for ecological restoration remain largely underexplored. Particularly, the objectives of exploiting waterborne epoxy resin for improving moisture retention and maintaining strength can be conflicting, and microscale analytical assessment revealing the modification mechanisms requires further clarification. Moreover, an improvement in structural characteristics does not necessarily provide suitable conditions for plant germination or growth, and this requires pilot-scale trials to distinguish and verify the vegetation potentials and biocompatibility.

This present study investigated the characteristics of triethylenediamine (TETA)-cured epoxy resin modification in cemented soil for potentially being deployed in slope stabilization and restoration. A cement-epoxy twofold stabilization system was developed and assessed by carrying out experimental tests on a series of specimens with varying content of binder and epoxy resin. Performance of the stabilization system was estimated by comparing its strength development, water stability, structural rigidity under simulated exposures, as well as alkalinity changes. Complementary characterization techniques, including X-ray diffraction (XRD), scanning electron microscopy (SEM), and thermogravimetric (TG) analysis, were employed to elucidate the modification mechanisms at a microstructural level. A pilot-scale test for vegetation was carried out to verify the influence. This paper ends with conclusions and recommendations for further work.

## 2. Materials and Methods

### 2.1. Materials

#### 2.1.1. Cement Binders

Three types of cement binders were used for stabilizing the soil sample, namely ordinary Portland cement (OPC), calcium sulfoaluminate cement (SAC), and Portland slag cement (PSC). The cements were obtained from three suppliers in China, and they all had a strength grade of 42.5. The OPC and PSC complied with standard GB 175-2023, whilst SAC conformed to GB/T 20472-2006. SAC is generally used as a replacement for Portland cement to reduce CO_2_ emissions [36]. Their main components derived by X-Ray fluorescence are given in Table 1. The SAC and PSC had comparatively lower contents of calcium oxides (CaO) and were expected to reduce the alkali release and, accordingly, mitigate the alkalinity level in cemented soil. The SAC was rich in alumina (Al_2_O_3_), for it involved Bauxite (Al_2_H_2_O_4_) as one main raw material in clinker calcination. Alternatively, the PSC had the highest componential content of SiO_2_ attributed to its addition of supplementary material of blast furnace slag. Apart from the presented oxides, the PSC was detected to have minimal other oxides of BaO, P_2_O_5_, K_2_O, Cr_2_O_3_, MnO, ZnO, SrO, and ZrO_2_ by mass fractions ranging in 0.01% to 0.5%. These oxides likely originated from the addition of slag, which is a byproduct of iron smelting. These characteristics, given in Table 1, indicate that the cements may present varying hydration and stabilization effects on soil.

#### 2.1.2. Triethylenediamine (TETA)-Cured Epoxy Resin

**Figure 1:**
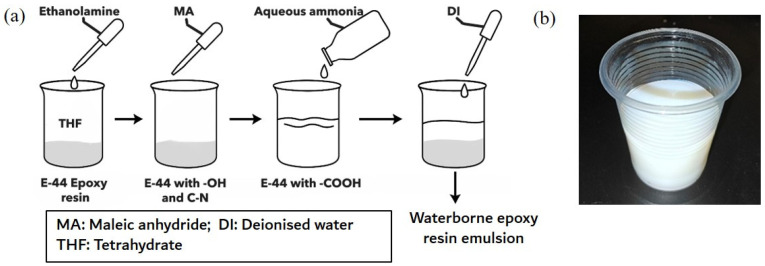
(**a**) Synthetic illustration of the preparation of epoxy resin emulsion, (**b**) Emulsified epoxy resin after adding triethylenediamine (TETA) curing agent.

A waterborne epoxy resin cured by triethylenediamine (TETA) was used for modifying the characteristics of soil and cemented soil. E-44 epoxy, supplied by Guangzhou Fufei Chemical Co., Ltd.(Guangzhou, China), was used, and the epoxy resin was emulsified to be waterborne and suitable for cemented soil addition. Reagents used for the process of emulsification included tetrahydrofuran, ethanolamine, maleic anhydride, and ammonium hydroxide solution, all supplied by Chengdu Kelong Chemicals Co., Ltd. (Chengdu, China). A controlled ring-opening polymerization was preliminarily present between the E-44 epoxy resin and ethanolamine that leveraged the primary hydroxyl group and introduced new C-N bonds in the epoxy resin. Maleic anhydride was added at a molecular ratio of 1:1 to ethanolamine, where the process of esterification between maleic anhydride and the hydroxyl group in tetrahydrofuran as a solvent occurred, and this introduced a carboxyl functional group (see Figure 1a). The mixture was then neutralized by aqueous ammonia to correspondingly form ammonium salts, after which deionized water was dropped into the admixture until a phase inversion from viscous liquid to emulsion was observed (see Figure 1b).

The curing agent for the prepared epoxy resin emulsion was made through a chain-extension reaction between the E-44 epoxy resin and TETA, where they were mixed at a mass ratio of 1:2. Anhydrous ethanol was added during the reaction as a diluent, which was removed by evaporation following completion of the reaction. The TETA-cured epoxy resin at its freshly mixed state was measured to have a rotational viscosity of approximately 7300 mPa·s at room temperature (20 °C).

#### 2.1.3. Soil

**Figure 2:**
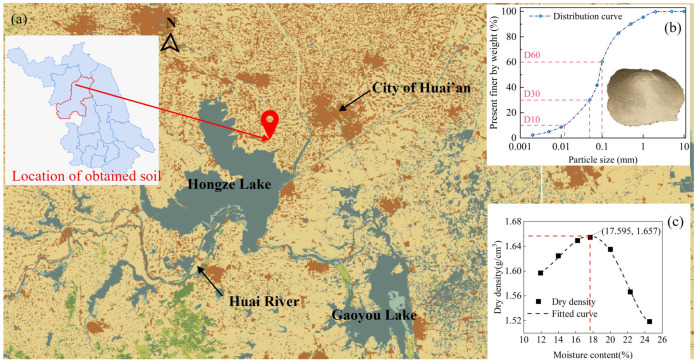
(**a**) Location of the obtained soil, (**b**) Curve of particle size distribution, (**c**) Relationship between the moisture content and dry density of the test soil.

Natural soil, which was obtained from near the Huaishu River bank situated in Huai’an City of Jiangsu Province, China, was used (see Figure 2a). The collected soil was air-dried, ground, and sieved (2.36 mm) to reduce interference to analysis from impurities or organic matter. Based on the particle size distribution test, the soil consisted mainly of sand in the presence of blocks of irregular shapes with minor clay content (see Figure 2b). The prepared soil sample was measured to have a water content (drying method) of 14.82% and a bulk density and apparent density of 1.27 g/cm^3^ and 1.35 g/cm^3^, respectively. A standard Proctor compaction test was carried out as per standard GB/T 50123-2019, and it was calculated that the optimum moisture content and maximum dry density of the sand were 17.59% and 1.66 g/cm^3^, respectively (see Figure 2c). The soil was measured to have a pH of 7.9. Table 2 summarizes key physical properties of the obtained natural soil.

### 2.2. Preparation of Soil Specimens

The soil specimens were prepared as per procedures recommended in the standard JGJ/T 233-2011 for cemented soil. Prior to mixing, the epoxy resin was first blended with its TETA curing agent at a mass ratio of 1:2, followed by the addition of water. The liquids were sufficiently blended until they formed a visually uniform mixture. After this, the liquid mixture was mixed with soil and cement using a machine mixer for 60 s at a lower speed of 140 r/min and a following higher speed of 300 r/min for 120 s. The water content of all soils was adjusted to 17.56%, which corresponded to their evaluated optimum moisture content (see Figure 2c). The samples were then placed into casting molds with sufficient machine vibration for compaction, and their surfaces were leveled. The specimens were demoulded after 48 h of initial curing, and curing was continued in controlled conditions (20 ± 2 °C and 90% relative humidity) in the laboratory until designated ages.

The unconfined compressive strength (UCS) of prepared soil samples was measured as per JGJ/T 233-2011 using cubic specimens of dimensions 70.7 mm. The loading was applied at a constant rate of 0.10 kN/s until failure. The pH of the prepared samples was measured according to GB/T 50123-2019. Specifically, extracted samples of cemented soil of 10 g were crushed and sieved, and were placed in a plastic graduated cylinder with 50 mL of added water. The container was manually shaken for 3 min and left still for 30 min to achieve better release of alkalis. The pH of the supernatant was measured using a calibrated portable pH meter. The specimens for each measurement comprised three duplicate samples.

### 2.3. Measurement Setup

#### 2.3.1. Change in Water Stability

The change in the water stability of the cement-stabilized soil resulting from the integration of water-retaining polymers was assessed by measuring its unconfined compressive strength (UCS). The coefficient of water stability ($\gamma_{ws}$) is defined as the ratio of the UCS of a soil specimen after being immersed in water for 24 h when its standard curing age reached 6 d and 27 d, respectively, to that of a specimen without water immersion at the ages of 7 d and 28 d, respectively. The UCS of the soil samples was measured with the electromechanical universal testing machine supplied by Instron (Norwood, MA, USA) as per standard JTG 3441-2024 and pertinent recommendations from CJ/T 486-2015. Loading was load-controlled with a constant rate of 0.1 kN/s until a compressive strain of >10% was achieved. The UCS ratio was calculated using Equation (1) as follows:

(1) $$\gamma_{s}=\frac{R}{R_{o}}\times 100$$

where $\gamma_{s}$ is the UCS ratio (%), R is the UCS of the tested soil specimen (MPa), $R_{o}$ is the UCS of the reference soil specimen (MPa).

The coefficient of water stability ($\gamma_{ws}$) of the tested soil specimen was calculated using Equation (2):

(2) $$\gamma_{ws}=\frac{R_{w}}{R}\times 100$$

where $\gamma_{ws}$ is the coefficient of water stability (%), $R_{w}$ is the UCS of the tested soil specimen after water immersion (MPa).

Consequently, the ratio of the coefficient of water stability ($\gamma_{e}$) was calculated using Equation (3):

(3) $$\gamma_{e}=\frac{\gamma_{ws}}{\gamma_{w0}}\times 100$$

where $\gamma_{e}$ is the ratio of the coefficient of water stability (%), $\gamma_{ws}$ is the coefficient of water stability of the tested soil specimen (%), $\gamma_{w0}$ is the coefficient of water stability of the reference specimen (%).

#### 2.3.2. Mass Loss of Cemented-Soil Under Repeated Wetting-Drying

The soil-cement losses induced by repeated wetting and drying of hardened cemented soil specimens were determined as per standard ASTM D559/D559M-15. After 28 days of standard curing, the soil specimens were submerged in tap water at room temperature for 5 h and removed, and the mass and water content were measured and recorded. After this, the specimens were placed in the oven at a constant temperature of 71 ± 3 °C for 42 h and removed, and the same measurements were also recorded. These two procedures constituted one cycle (48 h) of wetting and drying, and continued the procedure for 12 cycles. The soil-cement loss rate (*ω*) was calculated using the following Equation (4):

(4) $$\omega =\frac{m_{e-}m_{n}}{m_{e}}\times 100$$

where *ω* is the cement-soil mass loss rate (%), $m_{e}$ is the mass of the tested specimen before the wetting-drying cycle of *n* (g), $m_{n}$ is the mass of the tested specimen after the wetting-drying cycle of *n* (g).

#### 2.3.3. Ultraviolet (UV) Light Aging Test

The UV aging test was conducted following the standard ASTM G154-23. Specimens cured at 6 days were placed in a UV aging chamber with irradiance of 0.76 W·m^−2^·nm^−1^ at 340 nm and temperature of 50 °C for 24 h. After the simulated weathering exposure, the UCS of the specimens was measured. Deterioration caused by UV aging was calculated as the UCS change in the specimen.

#### 2.3.4. Mineralogical Composition and Microstructure Characteristics Analysis

Analytical tests, including X-ray diffraction (XRD), thermogravimetric (TG) analysis, and scanning electron microscope (SEM) techniques, were performed to evaluate the mineralogical composition change and microstructure characteristics of the cement-stabilized soil with polymer. Specifically, the XRD analysis was performed using the diffractometer (Rigaku, Japan) that emitted an X-ray source (40 kV and 40 mA) of 2θ scanning range of 5° to 90° and a scan increment rate of 2°/min. Retrieved soil samples were ground and sieved (0.15 mm) and oven-dried at 30 °C for 24 h until they reached a constant weight. Alternatively, the TG analysis was conducted using the Simultaneous Thermal Analyzer (PerkinElmer, Thane, MA, USA). The prepared samples (same as for the XRD) were heated at a temperature range of 30–1000 °C for a heating rate of 10 °C/min under a nitrogen atmosphere. Equation (5) was used to estimate the mass fraction ($m_{j}$, %) of phase *j* obtained from the TG curves:

(5) $$m_{j}=\frac{{dm}_{j}\times M_{j}}{n\times M_{H_{2}O}}$$

where ${dm}_{j}$ is the mass loss calculated from the TG data of phase *j* (%), $M_{j}$ is the molecular weight of phase *j* (g/mol), *n* is the number of water molecules of phase *j*, and $M_{H_{2}O}$ is the molecular weight of water (g/mol).

## 3. Results

Results of the modification of TETA-cured epoxy resin on both natural soil and cemented soil were presented and analyzed. A range of modified soils was considered, including varying epoxy resin and cement binder dosages. The study was aimed at developing and understanding the effects occurring and characteristics of TETA-cured epoxy resin modification in cemented soil and providing practical information. The present results have relevance to the further application of modified soil in porous vegetation concrete construction.

### 3.1. Influence of TETA-Cured Epoxy Resin Modification on Natural Soil

**Figure 3:**
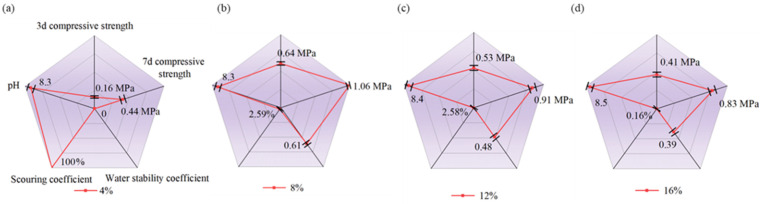
Influence of TETA-cured epoxy resin dosage on the changes in UCS, water stability, and alkalinity of natural soil modification: (**a**) 4%, (**b**) 8%, (**c**) 12%, and (**d**) 16%.

Figure 3 presents the measured UCS strength, water stability, scouring coefficient, and pH of natural soil specimens stabilized with only TETA-cured epoxy resin. The specimens were prepared with varying epoxy resin dosages of 4–16% by mass of soil and at an increase interval of 4%. Experimental results indicate that the incorporation of the TETA-cured epoxy resin was effective in enhancing strength and water stability, whilst the enhancement was remarkably pertinent to the epoxy resin dosage.

A maximum UCS of 1.06 MPa was achieved at 7 days for the specimen with 8% epoxy addition (see Figure 3b), which was a remarkable enhancement in comparison with the negligible compressive resistance presented by pure soil. The natural soil had an effective size D_10_ of 0.012 mm and a uniformity coefficient C_u_ of 8.3, manifesting its main composition was sand with a broad distribution of particle size (see Figure 2b). For this reason, the soil was incohesive, attributed to the fact that it mainly consisted of granular sand particles. Alternatively, the epoxy resin, as a high-molecular polymer, can preliminarily encapsulate sand particles due to van der Waals interactions and electrostatic attractions [32,33,34,37]. These encapsulated sand particles were then bonded together by interfacial crosslinking, leading to increased adhesion and improved UCS. However, it was also observed that the UCS decreased when the epoxy resin content exceeded 8%. Specifically, specimens with 12% and 16% epoxy addition exhibited reduced UCS of 0.91 MPa and 0.83 MPa, respectively (see Figure 3c,d). This was likely due to the excessive waterborne epoxy mitigating grain frictions and subsequently reducing UCS.

Similar trends in the change in water stability coefficient were observed. Specimens with 8% epoxy addition exhibited the highest water stability coefficient of 0.61. With the increase in added epoxy dosage to 12% and 16%, the water stability coefficient decreased to 0.48 and 0.39, respectively, manifesting a destabilization effect from excessive presence (>8%) of waterborne epoxy resin. Alternatively, a higher dosage of TETA-cured epoxy resin was found to reduce the soil’s mass loss from simulated water erosion. At 4% epoxy addition, the specimen disintegrated after scouring, yielding a rate of mass loss of 100% (see Figure 3a). However, when the epoxy addition increased to ≥8%, the mass loss notably reduced, ranging between 2.58% and 0.16%.

The alkalinity of the soil also changed after the TETA-cured epoxy resin stabilization. The obtained natural soil sample originally had a pH of 7.9, whilst the alkalinity increased to between 8.3 and 8.5 after epoxy resin incorporation. This was likely caused by the TETA curing agent as well as the polymerization and emulsification process that involved using aqueous ammonia for neutralization (see Figure 1a). Although organic and inorganic binders (for instance, cement and lime) can both be deployed for soil stabilization, waterborne epoxy resin offers a distinct advantage in maintaining soil alkalinity to relatively constant levels. This is particularly important for its application in porous vegetation concrete [4]. However, results implied that incorporating only TETA-cured epoxy resin could contribute a limited level of stabilization.

### 3.2. Identifying the Effect of TETA-Cured Epoxy Resin Modification on Cemented Soil

#### 3.2.1. Optimization of the Cement-Epoxy Twofold Stabilization System

A twofold stabilization system of using Portland cement and TETA-cured epoxy resin was investigated. Table 3 presents the specimen mix proportion with varying content of OPC 42.5 of 4%, 6%, and 8% by mass in combination with waterborne epoxy resin of 0, 1% and 2% by mass, respectively. The soil itself had a moisture content of 17.56% (see Figure 2c). Additional water equal to the cement mass was introduced to achieve a water-to-cement ratio of 1 as per standard JGJ/T 233-2011. The specimens were named after their cement and epoxy content. For example, C4E1 represents a cement content of 4% and an epoxy resin content of 1%.

**Figure 4:**
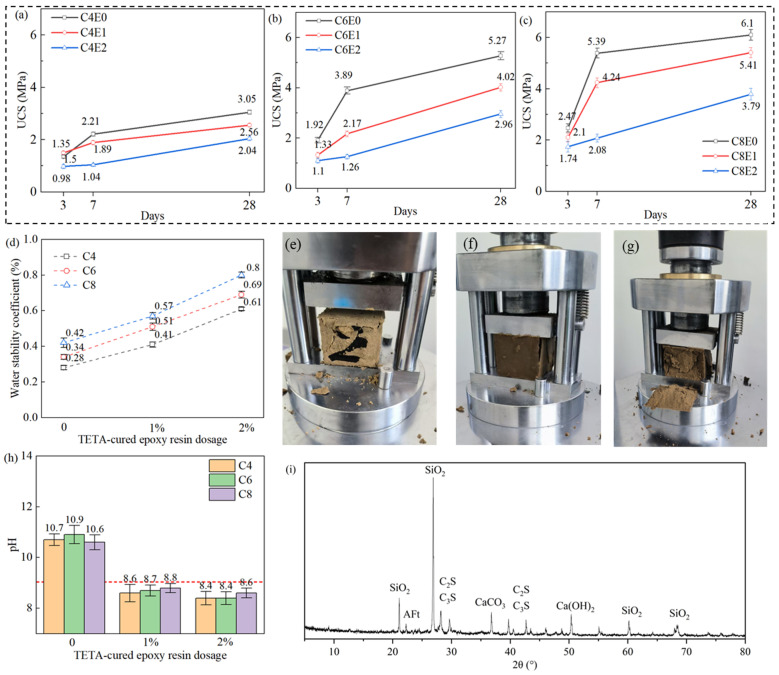
Influence of TETA-cured epoxy resin on performance of cemented soil: (**a**–**c**) influence of epoxy resin dosage in the UCS development, (**d**) change on water stability coefficient, (**e**) unconfined compressive failure of C8E2 before water immersion, (**f**,**g**) unconfined compressive failure of C8E2 specimens after water immersion, (**h**) change in pH (red dash line: pH = 9.0 as per JC/T 2557-2020), (**i**) representative XRD results from C8E2.

Figure 4 presents test results indicating the influence of the cement-epoxy twofold stabilization system. As depicted in Figure 4a–c, cement was exceptionally effective in stabilizing natural soil. This was reflected by the notably increased UCS of the tested specimen, which, for instance, C8E0 achieved 6.1 MPa at 28 days. The stabilization effectiveness of cement was remarkable in comparison with that of solely TETA-cured epoxy resin. For instance, C8E0 of 8% cement addition had a UCS of 5.39 MPa at 7 days (see Figure 4c), and this was ~5 times larger than that of specimens with 8% TETA-cured epoxy resin (see Figure 3b). This effectiveness difference was attributed to the stabilization of cement, which relied on chemical hydration of the clinkers. Alternatively, the incorporation of the TETA-cured epoxy resin reduced the UCS for cemented soil. Specifically, for specimens with varying OPC content, 1% epoxy addition typically reduced the UCS to 55–85%, and 2% epoxy addition reduced it to 30–45%. This was likely induced by the film-forming capacity of epoxy resin that encapsulated particles from cement hydration and hindered this process.

However, this research focused on soil stabilization for porous vegetation concrete that was potentially applied for slope stabilization and green-growing. Waterborne epoxy resin was still essentially demanded for controlling alkalinity and maintaining water stability. The obtained experimental results confirmed the necessity of the investigated cement-epoxy twofold stabilization system. Cement stabilization, although very effective in improving soil strength, yielded a pH level that might be harmful for plant germination and growth. As per standard JC/T 2557-2020, a pH < 9.0 (see the dashed horizontal line in Figure 4h) is recommended where the alkalinity level in cement-stabilized soil reaches pH > 10.6. Alternatively, incorporation of the TETA-cured epoxy resin reduced the pH of cemented soil to <8.8. These results indicate that waterborne epoxy resin effectively suppressed the formation of a high-alkali environment caused by cement hydration through forming a polymeric film and subsequently restricting the release or migration of alkalis.

Moreover, the cement-epoxy twofold stabilization system remarkably enhanced the water stability of cemented soil. A highest water stability coefficient of 0.8 was achieved by specimen C8E2 (see Figure 4d), indicating exceptional structural stability when subjected to water immersion. This water stability was superior to that of both sole OPC and TETA-cured epoxy resin. Cemented soil without epoxy exhibited a water stability coefficient of 0.42 (Specimen C8E0), whilst for specimens with TETA-epoxy resin modification, the measured maximum value was 0.61 (see Figure 3). The failure modes of specimens using cement-epoxy twofold stabilization for testing water stability varied after immersion. Specimen C8E2, as a representative, had a failure mode of propagated cracks, which is similar to the typical failure mode of concrete cubes when measuring compressive strength (see Figure 4e). After water immersion, the specimens had a failure mode of the side face delamination (see Figure 4f,g). This revealed the importance of waterborne epoxy resin in enhancing water stability. The stability of structural integrity is vital for porous vegetation concrete because the restoration confronts moisture impact from precipitation as well as irrigation.

Figure 4i depicts representative XRD peaks with quartz (SiO_2_) being identified as a dominant crystalline phase from the source of sand. Feldspar minerals contributed several secondary reflections, whereas cement hydration products such as portlandite produced overlapping peaks in the higher angle region of 2θ of around 50°. The epoxy resin was not manifested for its being amorphous and non-crystalline. It was noticed from the XRD results that the incorporation of organic polymer binder did not result in remarkable componential deviation for cemented soil specimens with varying epoxy dosage.

#### 3.2.2. Microstructural and Mineralogical Analysis

**Figure 5:**
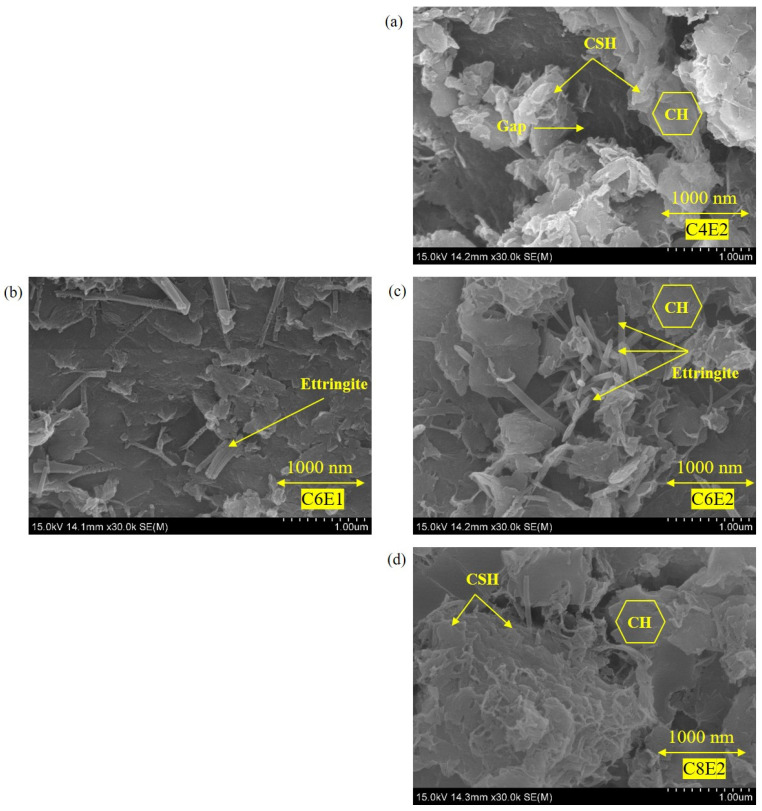
SEM (30,000×) images of cemented soil with TETA-cured epoxy resin modification: (**a**) C4E2, (**b**) C6E1, (**c**) C6E2, and (**d**) C8E2.

Although the incorporation of TETA-cured epoxy resin did not contribute to chemically formation of hydration minerals, it was found to affect the microstructural and mineralogical characteristics of cemented soil stabilization. This was reflected by the SEM results by comparing the effects of the twofold stabilization system. As depicted, the influence of the cement binder dosage was dominant. This was manifested by the microstructural characteristic changes from specimens C4E2, C6E2, and C8E2 (see Figure 5a,c,d). Specifically, C4E2 presented relatively unconsolidated structures with pores distinguished between these irregular aggregate particles and cement hydration products, which likely offered limited densification. When the cement content increased, C6E2 and C8E2 exhibited remarkably densified structures with visually reduced porosity and improved interfacial bonding between particles. However, different phase-products originated from cement hydration between C6E2 and C8E2 were distinguished. C6E2 was detected to exhibit more needle-like ettringite, whilst C8E2 mostly had agglomerated fibrous C-S-H gels and presented a superior structure, only depicting minor voids. This indicated that C6E2 was at a more retarded cement hydration stage than C8E2 by the time this SEM observation was carried out (7 days). This conforms to the obtained UCS development presented in Figure 5a,c,d. In this study, the pore structure analysis is mainly based on qualitative analysis from SEM observations. An introduction of Micro-CT will allow the pore structure and connectivity to be captured three-dimensionally [38], which will facilitate a more accurate analysis of the aeration and water-retention properties.

**Figure 6:**
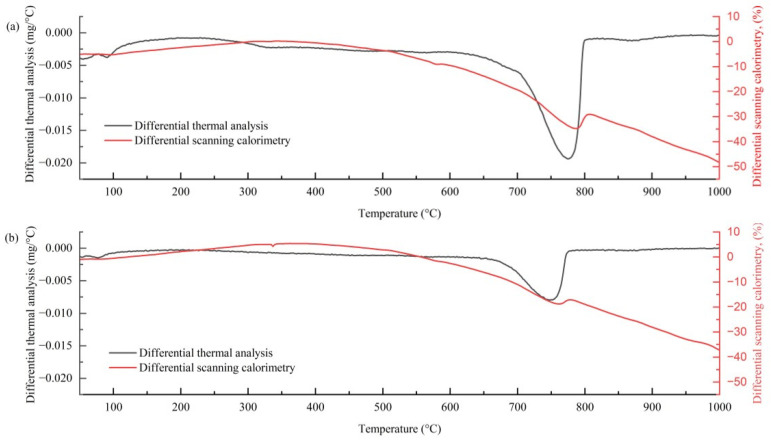
Thermal mass loss rates of cemented soil with TETA-cured epoxy resin modification: (**a**) C6E1, (**b**) C6E2.

The thermal decomposition results of TG/DTG also confirmed the influence of the twofold stabilization system on cement hydration. Figure 6 presents the DTG and DSC (Differential Scanning Calorimetry) curves of C6E1 and C6E2. A major mass loss concentrated in the heating range of 600–800 °C, primarily associated with the decomposition of Ca(OH)_2_ and likely some CaCO_3_ from cement hydration products. It can be seen that C6E1 exhibited a deeper trough, indicating greater mass loss of calcium hydroxide. This also confirmed that increased epoxy resin content impeded cement hydration and conformed to the SEM images in Figure 5b,c. Apart from this, typical decomposition trends of C6E1 and C6E2 were similar, with slightly varied minor weight loss occurring in the heating range of 150–400 °C, representing decomposition of the epoxy resin and TETA curing agent.

#### 3.2.3. Durability Performance of the Cement-Epoxy Twofold Stabilization System

**Figure 7:**
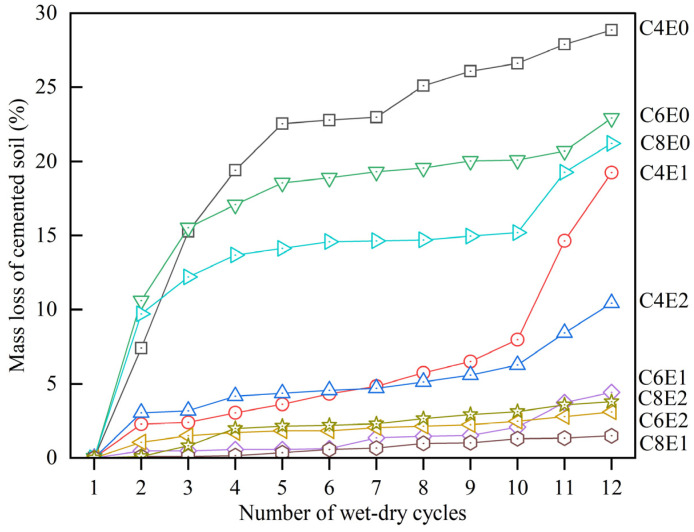
Cumulative mass loss rate of specimens after cyclic wetting-drying cycles.

Stabilized soil in porous vegetation concrete confronts durability impacts from cyclic evaporation and precipitation in combination with long-term outdoor UV weathering [39]. For this reason, the durability performance of the cement-epoxy stabilization system is vital. This research examined the strength and stability performance of stabilized soil by deploying simulated wetting-drying and accelerated UV exposure. Figure 7 presents the cumulative mass loss rate from 12 continuous wetting-drying cycles. An increase in cement content contributed to mitigating the mass loss. Specifically, when the content of added cement increased from 4% to 8%, the mass loss rate reduced from 27.89% to 13.69%. With the incorporation of TETA-cured epoxy resin, the mass loss was improved to typically <5%. Specimens C4E1 and C4E2, however, exhibited an emergently increased mass loss rate after 10 cycles. This implied the importance of sufficient cement binder presence within the twofold soil stabilization system, for it provided a comparatively rigid strengthening structure for the unconsolidated soil. Consequently, it can be speculated that the combined adjustment of cement and waterborne epoxy resin could synergistically demonstrate a pronounced effect in mitigating the structure deconstruction resulting from cyclic moisture content variation.

Alternatively, cemented soil with TETA-cured epoxy resin modification was observed to exhibit notable strength loss after exposure to accelerated UV aging. Table 4 gives the rate of UCS reduction for different stabilization specimens. Cemented soil presented a strength decrease between 5% and 7% after the UV stimulation at 50 °C for 24 h. This was likely caused by sample dehydration. However, for cemented soil with TETA-cured epoxy resin, this decreasing trend enlarged to an extent between 10% and 15%. As an organic binder, epoxy resin was prone to oxidative photodegradation under UV irradiation that weakened the polymeric film due to the combined action of light and oxygen, and consequently impacted the interparticle bonding. It is worth noting that supplements of UV stabilizers or antioxidants are reported to be able to improve the outdoor service behavior of polymers such as epoxy resin. For instance, Nikafshar et al. [40] reported that encapsulated halloysite nanotubes with organic UV-stabilizers and lignin improved the UV-stability of epoxy coating. However, some types of UV stabilizers may raise concerns about the bioaccumulation of toxicity [41] which can be problematic for this study’s application of plant restoration.

Overall, the reduction in the cement-epoxy twofold stabilization system in soil was moderate and acceptable for porous vegetation concrete. Typically, well-designed porous vegetation concrete possesses a compressive strength of ≥10 MPa at 28 days [42,43], whilst the cemented soil, as an upper layer mainly for germination, is generally ≥1 MPa [1,44,45,46]. Although the UCS of cemented soil reduced after UV irradiation, they are still sufficient (see Table 4) except for C4E2 due to its lower cement and higher epoxy content. However, the coupled impact of cyclic wetting-drying and UV irradiation may intensify this strength reduction [47,48] and deserves further research attention with respect to providing a more realistic assessment of long-term durability.

### 3.3. Influence of Cementitious Binder Type

**Figure 8:**
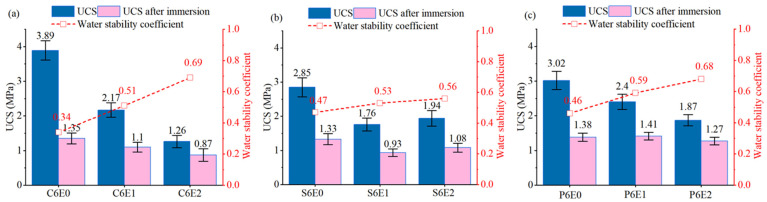
Influence of cement binder type on TETA-cured epoxy resin modification at 7 days: (**a**) OPC, (**b**) SAC, (**c**) PSC.

Apart from Portland cement, there are other types of cement binders available, which were developed for reducing the use of CaO compositions in the clinker. These binders have potential for alkali reduction. This research examined the use of SAC and PSC (see Section 2.1.1) as alternatives to OPC in the cement-epoxy twofold stabilization system. These specimens all had a cement content of 60 kg/m^3^ and varying epoxy resin content of 10 kg/m^3^ and 20 kg/m^3^, respectively, with corresponding TETA for curing (see Table 5). Figure 8 presents the measured 7-day UCS and water stability coefficient, which exhibited the ability to maintain structural stability after being immersed in water. Specimens with SAC cement and PSC cement exhibited lower UCS at 7 days in comparison with those with OPC. Specifically, they had UCS of 2.85 MPa and 3.02 MPa, which were 73% and 78% of that of the OPC specimens when they all had no incorporation of TETA-cured epoxy resin. For OPC cemented soil, TETA-cured epoxy resin reduced the UCS, whilst this reduction was more severe when the epoxy resin dosage increased from 1% to 2% (see Figure 4b). The UCS of OPC specimens with epoxy resin (C6E1 and C6E2) only received 30–50% of that of C6E0. However, for SAC and PSC cemented specimens, this relationship was different. They exhibited less reduced UCS resulting from the epoxy resin incorporation that was 60–80% of cemented soil (see Figure 8b,c). Notably, for SAC cement, specimen S6E2 with 2% epoxy resin had a higher UCS in comparison with S6E1 with 1% epoxy. This was the only type of cement that exhibited increased UCS with increased epoxy dosage among the investigated three types, although this UCS was still lower in comparison with S6E0. This variation was attributed to the difference in hydration mechanisms of different cement type.

The early-stage strength development of ordinary Portland cement mainly originated from the hydration of C_3_S and C_2_S that formed C–S–H gel and Ca(OH)_2_. When waterborne epoxy resin was incorporated, its film-forming behavior inhibited ion migration and retarded C_3_S and C_2_S dissolution. This thereby impeded the initial hydration and resulted in a pronounced reduction in 7-day UCS, as depicted in Figure 8a. Different from OPC, the early-stage strength development of calcium sulfoaluminate (SAC) cement is predominantly derived from the hydration of C_4_A_3_S and production of AFt and AH_3_ [49,50]. Although the presence of the organic phase of epoxy resin could potentially hinder ion diffusion, the inherently high reactivity of SAC cement and the abundant formation of AFt partially compensated for this [51,52]. Moreover, the hydration process of SAC cement was accompanied by the transformation of AFt to AFm, which contributed to densifying the microstructure [53,54]. Formation of the relatively stable aluminate phases was speculated to mitigate the adverse influence of the TETA-cured epoxy resin. Consequently, the reduction in UCS at 7 days for SAC cement specimens was relatively moderate in comparison with OPC ones (see Figure 8a,b). It is worth noting that variable curing accelerators for the epoxy system are available [55] if applications where slopes require immediate stability occur. Alternatively, different TETA/epoxy ratios contribute to recovering this early-stage strength reduction due to the retardation of cement hydration, although the water retention behavior may also be altered accordingly.

For Portland Slag Cement (PSC), the increased content of granulated blast furnace slag resulted in relatively slow PC clinker hydration activation [54,56,57]. Attributed to this hydration characteristic of PSC, the influence of the waterborne epoxy resin on cemented soil strength development was the least impactful among the three tested cement binders (see Figure 8). For a relatively slow and long-lasting hydration process, the mutual interaction between C–S–H and the epoxy resin membrane was speculated to create a complementary interface that increased particle bonding and enhanced structural integrity. Overall, the influence of TETA-cured epoxy resin on different cement stabilization dominantly depended on their type and rate of hydration reaction.

**Figure 9:**
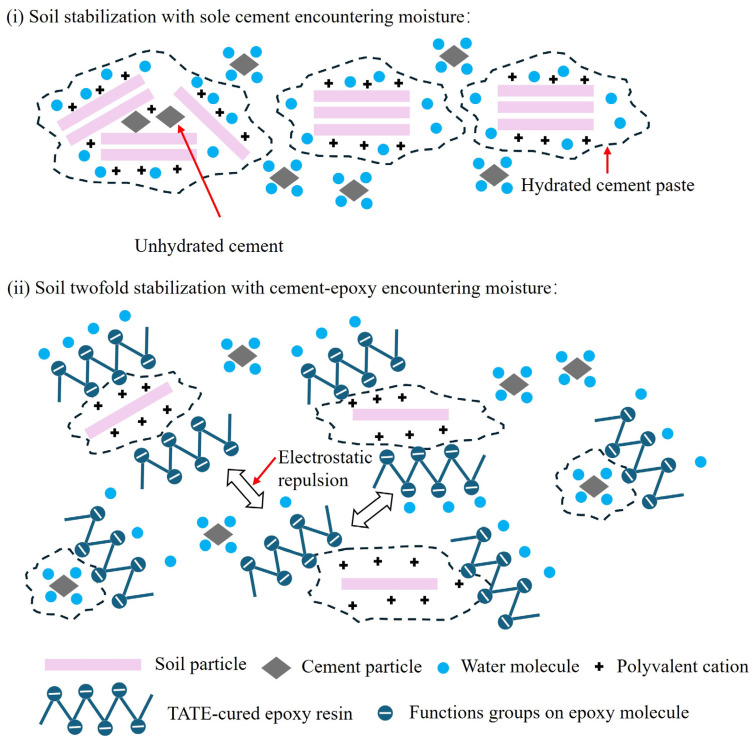
Schematic illustration of the influence of the modification mechanism difference in cemented soil with TETA-cured epoxy resin: pore structure refinement and water retention improvement.

For all tested specimens with different cement types, the addition of epoxy resin induced an improved water stability coefficient. However, the SAC cement exhibited the lowest improvement. For instance, the water stability coefficient specimen S6E2 was 0.56 (see Figure 8b). This means that the UCS after immersion was only approximately 50% of its strength prior to wetting. It is worth noting that cemented soil specimens with PSC manifested higher UCS after immersion in comparison to those of OPC and SAC. The retarded hydration reaction by granulated blast furnace slag was progressively activated by Ca(OH)_2_ [54,58]. This progress worked synergistically with the membrane formed by the waterborne epoxy resin, contributing to the development of a comparatively dense microstructure. These structural characteristics impeded the likely dissolution of cement hydration products or water penetration and consequently achieved a rational balance between the strength retention and water stability enhancement (see Figure 8c). This is beneficial for stabilization and restoration of slopes where they may be exposed to frequent moisture content variation. Figure 9 schematically illustrates the influence of the mechanics of TETA-cured epoxy resin in modifying cemented soil by refining the pore structures and improving water retention capacity. Although the addition of waterborne epoxy resin improves the engineering and ecological performance of cemented soil, a comprehensive life-cycle assessment and cost–benefit analysis would further strengthen its application potential.

### 3.4. Plant Growth Performance and Potentials for Porous Vegetation Concrete

The vegetation test was carried out in order to assess the revegetation ability of the cemented soil with waterborne epoxy resin. The vegetation test was implemented in an outdoor environment to simulate natural germination and growing conditions for assessing the plant growth performance. Specimens with 6% OPC addition were used, i.e., C6E0, C6E1, and C6E2. A 30-day experiment was carried out at the campus of Hohai University located in Jintan District of Changzhou, China (119°57′ E, 31°67′ N; WGS84 coordinate system) from mid-May to mid-June 2025. Meteorological data recorded average monthly minimum and maximum temperatures of 17–28 °C in May and 20–31 °C in June, with total precipitation measuring 134.9 mm and 85.5 mm, respectively [59]. Cubic specimens of 100 mm of the cemented soil were prepared and placed in a plastic container serving as the seeding substrate. Three species of plants were selected to be seeded on the cemented soil, namely, tall fescue (*Lolium arundinaceum*), Indigo Bush (*Amorpha fruticosa* L.), and Lespedeza bicolour (*Lespedeza bicolor Turcz.*). Approximately 100 mixed seeds of the three plants were uniformly distributed on top of the cemented soil specimens. A layer of nutrient soil of ~2 mm thickness was spread covering the cemented soil for sheltering and protecting seedlings. Throughout the vegetation test, the plants were supplemented with tap water irrigation twice daily when there was no rainfall precipitation. Morphological behavior of the plants, including their germination rate, germination time, and leaf extension, was measured and recorded at 5-day intervals.

**Figure 10:**
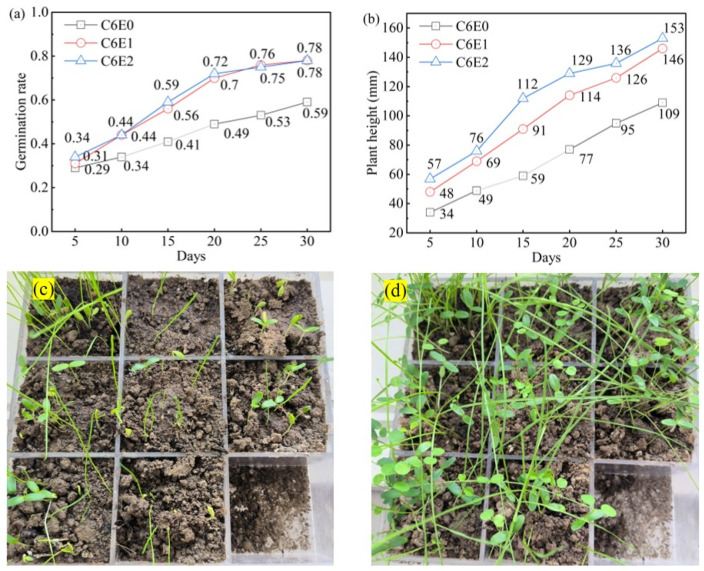
Vegetation performance on cemented soil with TETA-cured epoxy resin: (**a**) Germination rate, (**b**) Change in plant height, (**c**) Vegetation performance of C6E2 after 5 days, and (**d**) Vegetation performance of C6E2 after 30 days.

As shown in Figure 10, the incorporation of waterborne epoxy resin remarkably improved the vegetation performance on OPC cemented soil, manifested by enhanced germination rate (see Figure 10a,c) and visually increased biomass and leaf extension (see Figure 10b,d). The germination rate of all cemented soil specimens exhibited an increasing trend during the 30-day experiment. Specifically, C6E0 achieved a germination rate of 0.59 at 30 days, whilst for C6E1 and C6E2 specimens, the inclusion of TETA-cured epoxy resin increased the germination rate to 0.76 and 0.78, respectively. This indicated that waterborne epoxy resin effectively mitigated the adverse impact of alkali release and diffusion on plant germination. Moreover, it was noticed that during the early stage of observation time of between 5 days and 15 days, the germination rate of C6E1 and C6E2 exhibited more remarkably increased trends in comparison with C6E0. This was likely contributed, to some extent, by the fact that the moisture level was better maintained by the incorporation of waterborne epoxy resin. By the end of the 30-day growth experiment, the leaf extension of C6E1 and C6E2, which was quantified by measuring the shoot height of plants, increased approximately 34% and 40%, respectively, in comparison with C6E0.

Typically, the modification of TETA-cured epoxy resin on cemented soil was exceptional in improving seed germination and early-stage plant growth. This predominantly relied on the polymeric membrane sealing effect that reduced the diffusion of soluble alkalis (see Figure 4h). Meanwhile, the membrane sealing reduced water evaporation and facilitated water content maintenance, which produced a more humidified condition for vegetation growth. Moreover, the modified pore structure characteristics, as revealed from the SEM images in Section 3.2.2, provided a more rational substrate that allowed fundamental aeration and provided voids for the propagation of plant root systems. However, there are many factors that may influence plant growth and survival apart from the characteristics of the growing substrate (i.e., the cemented soil for this case). A 30-day pilot-scale trial is insufficient for assessing long-term plant survival or root system development. The layer of fertilizer soil of ~2 mm thickness used for covering cemented soil (see Figure 10c) supported plant initial growth. This theoretically allowed nutrients to diffuse from the fertilizer layer to the growing substrate. Alternatively, in a case where the fertilizer is mixed with the substrate, the effectiveness of nutrient leaching might be affected by the epoxy membrane, which warrants further research attention.

Moreover, the mutual effects exerted between plant root propagation and soil stabilization warrant further research outputs. Although root reinforcement is widely recognized as one of the most relevant for slope stabilization [60], its action on this designed twofold soil stabilization system of cement and epoxy resin may vary. It may cause degradation of the strengthening because of root-induced cracking and deconstruction to the modified pore structures, as schematically illustrated in Figure 9.

## 4. Discussion and Conclusions

This study evaluated the influence of TETA-cured epoxy resin modification on both natural soil and cemented soil by analyzing their mechanical, durability, and ecological performance. The modification mechanisms on cemented soil with varying binders of OPC, SAC, and PSC were elucidated through multi-scale analyses. The main conclusions drawn from this study are summarized as follows:

1. The cement-epoxy twofold stabilization system provided exceptional effects on modifying the soil for porous vegetation concrete. The TETA-cured epoxy resin offers soil stabilization predominantly from embracing sand particles and electrostatic crosslinking. Test indicates that an increase in the epoxy resin when exceeding 8% reduced stabilization and consequently restricts its application for porous vegetation concrete. The cement-epoxy twofold stabilization system induced reasonably reduced UCS and increased water stability in comparison with solely the cement binder. XRD results indicated that TETA-cured epoxy resin did not contribute to chemical variation in cement hydration, whilst SEM and TG/DTG results revealed that it retarded cement hydration and improved particle bonding. This change is responsible for the impeded UCS development and increased water stability.
2. The cement-epoxy twofold stabilization system was effective in enhancing structural stability exposed to repeated moisture content variation induced by cyclic wetting/drying. The cement content contributed to a detrimental structure, whereas waterborne epoxy resin mitigated mass loss by crosslinking the particles together. The twofold stabilization system was susceptible to oxidative photodegradation under UV irradiation, exhibiting increased UCS reduction rate from <7% to between 9% and 16%.
3. Cementitious binder type affected the modification effect of the cement-epoxy twofold stabilization system. Specimens with SAC and PSC cement exhibited lower UCS at 7 days in comparison with those with OPC, whilst cemented soil specimens with PSC manifested higher UCS after water immersion than those with OPC and SAC. The retarded hydration reaction by granulated blast furnace slag was progressively activated by Ca(OH)_2_ and worked synergistically with the membrane formed by the waterborne epoxy resin, contributing to the development of a comparatively dense microstructure. These structural characteristics impeded the likely dissolution of cement hydration products or water penetration and consequently achieved a rational balance between the strength retention and water stability enhancement.
4. Incorporation of the TETA-cured epoxy resin effectively reduced the alkalinity of cemented soil from a pH of >10.6 to ~8.8. This predominantly promoted the germination and growth of vegetation on cemented soils, with improved germination rate at 30 days from 0.59 to approaching 0.8, and visually extended leaves and biomass. This biocompatibility enhancement, although only short-term, is being investigated and was partially attributed to the membrane sealing of epoxy resin that reduced water evaporation and facilitated moisture content maintenance in combination with the modified pore structure characteristics. These make TETA-cured epoxy resin modification suitable for cemented soil on completed slope restoration.

The following topics are outlined as recommendations for further research. They may be considered as a next step of work for conducting related investigations aiming at development of stabilized soil for porous vegetation concrete: (i) a more holistic assessment of the cement–epoxy stabilization performance under coupled impact of UV and cyclic wetting–drying, as well as the incorporation of UV stabilizers or antioxidants to improve long-term outdoor service performance, (ii) the use of epoxy curing promoters and adjustments to the TETA/epoxy ratio for optimizing the early-stage strength development of cemented soil, and (iii) field-scale ecological validation on engineering slope stabilization for a full growing season (6–12 months) to assess the vegetation adaptability and structural rigidity in real restoration environments.

## Figures and Tables

**Table 1:** Oxide analysis of cements derived from X-ray fluorescence (mass fraction, %).

	SiO_2_	Al_2_O_3_	Fe_2_O_3_	CaO	MgO	SO_3_	Na_2_O	TiO_2_
OPC	14.61	8.47	5.30	66.27	1.41	1.70	1.03	0
SAC	7.23	18.60	4.30	45.30	1.35	12.50	0	0.87
PSC	26.76	12.00	2.00	47.12	6.06	4.19	0	0.38

**Table 2:** Key physical properties of the obtained soil sample.

Bulk Density (g/cm^3^)	Natural Water Content (%)	Optimum Water Content (%)	pH
1.35	14.8%	17.56%	7.9

**Table 3:** Specimens for optimizing the cement-epoxy twofold stabilization system for soil (kg/m^3^).

	Soil *	Cement	Water **	Epoxy Resin	TETA Curing Agent
C4E0	1000	40	40	0	0
C4E1	1000	40	40	10	3.3
C4E2	1000	40	40	20	6.7
C6E0	1000	60	60	0	0
C6E1	1000	60	60	10	3.3
C6E2	1000	60	60	20	6.7
C8E0	1000	80	80	0	0
C8E1	1000	80	80	10	3.3
C8E2	1000	80	80	20	6.7

* 17.56% water content (optimum moisture content). ** Including water contributions from epoxy resin (~60% water content) and TETA curing agent (~40% water content).

**Table 4:** Rate of UCS reduction after being exposed to UV weathering.

Specimen	C4E0	C4E1	C4E2	C6E0	C6E1	C6E2	C8E0	C8E1	C8E2
UCS after UV (MPa)	2.1	1.72	0.88	3.65	1.86	1.06	4.99	3.71	1.88
UCS reduction rate	5%	9%	15%	6%	14%	16%	7%	13%	10%

**Table 5:** Specimens with varying cement type (kg/m^3^) and TETA-cured epoxy resin.

	Soil *	Cement Type	Cement	Water **	Epoxy Resin	TETA Curing Agent
C6E1	1000	OPC	60	60	10	3.3
C6E2	1000	OPC	60	60	20	6.7
S6E1	1000	SAC	60	60	10	3.3
S6E2	1000	SAC	60	60	20	6.7
P6E1	1000	PSC	60	60	10	3.3
P6E2	1000	PSC	60	60	20	6.7

* 17.56% water content (optimum moisture content). ** Including water contributions from epoxy resin (~60% water content) and TETA curing agent (~40% water content).

## Data Availability

The original contributions presented in this study are included in the article. Further inquiries can be directed to the corresponding author.
